# Pregnane X Receptor‒4β‐Hydroxycholesterol Axis in the Regulation of Overweight‐ and Obesity‐Induced Hypertension

**DOI:** 10.1161/JAHA.121.023492

**Published:** 2022-03-01

**Authors:** Roosa Rahunen, Outi Kummu, Vesa Koivukangas, Heidi Hautajärvi, Jukka Hakkola, Jaana Rysä, Janne Hukkanen

**Affiliations:** ^1^ Research Unit of Internal Medicine University of Oulu Finland; ^2^ Biocenter Oulu University of Oulu Finland; ^3^ Medical Research Center Oulu Oulu University Hospital and University of Oulu Finland; ^4^ Research Unit of Biomedicine Pharmacology and Toxicology University of Oulu Finland; ^5^ Department of Surgery Oulu University Hospital and University of Oulu Finland; ^6^ Admescope Ltd. Oulu Finland; ^7^ Now with Finnish Customs Laboratory Finland; ^8^ School of Pharmacy Faculty of Health Sciences University of Eastern Finland Kuopio Finland

**Keywords:** 4β‐hydroxycholesterol, blood pressure, liver X receptor, obesity, pregnane X receptor, High Blood Pressure, Hypertension, Obesity, Translational Studies

## Abstract

**Background:**

Mechanisms mediating hypertensive effects of overweight and obesity have not been fully elucidated. We showed previously that activation of pregnane X receptor (PXR) by rifampicin elevates 24‐hour blood pressure (BP) and plasma 4β‐hydroxycholesterol (4βHC), agonist for liver X receptor (LXR).

**Methods and Results:**

In combined “PXR activation data set” (n=62) of 4 clinical trials, 1 week rifampicin dosing increased office systolic BP (SBP) by 3.1 mm Hg, DBP 1.8 mm Hg, and mean arterial pressure 2.2 mm Hg in comparison with placebo (*P*<0.01). Plasma 4βHC had negative correlation with SBP both in rifampicin (*r*=−0.46, *P*=0.0002) and placebo (*r*=−0.45, *P*=0.0003) arms, although 4βHC was elevated >3‐fold by rifampicin. In “non‐intervention data set” (n=102) of patients with obesity and healthy volunteers (body mass index, 19.2–55.2 kg/m^2^), 4βHC had negative correlations (*P*<0.00001) with office SBP (*r*=−0.51), diastolic BP (*r*=−0.50), and mean arterial pressure (*r*=−0.54). Lean women had higher 4βHC than men, with increasing weight repressing 4βHC (*r*=−0.62, *P*<0.00001) in both sexes. In multiple linear regression analysis, the only statistically significant predictor for SBP was 4βHC. Six‐day PXR agonist dosing elevated SBP in rats (n=7–8/group). PXR activation elevated 4βHC and after PXR agonist was withdrawn and elevated 4βHC was left to act alone, SBP was reduced on days 7 to 14 in comparison with control rats.

**Conclusions:**

PXR activation elevates SBP. Elevated circulating 4βHC lowers SBP in rats, and higher 4βHC is an independent predictor of lower SBP in humans. PXR‐4βHC‐LXR is novel BP‐regulating pathway deregulated in overweight and obesity by repressed 4βHC, with implications for sex‐specific BP regulation.

**Registration:**

URL: https://www.clinicaltrials.gov; Unique identifiers: NCT00985270, NCT01293422, NCT01690104, NCT02329405, and NCT01330251.

**Non‐Standard Abbreviations and Acronyms**
4βHC: 4β‐hydroxycholesterol
CYP: cytochrome P450
LXR: liver X receptor
PCN: pregnenolone 16α‐carbonitrile
PXR: pregnane X receptor
RYGB: Roux‐en‐Y gastric bypass surgery

**Clinical Perspective**

**What Is New?**

- We provide evidence for the existence of a new blood pressure‐regulating circuit involving pregnane X receptor (PXR), plasma 4β‐hydroxycholesterol (4βHC), and liver X receptor (LXR).
- PXR activation elevates blood pressure and the PXR‐regulated circulating 4βHC, an agonist for LXR, is suggested to counteract the hypertensive effect of PXR activation.
- Lean women have higher plasma 4βHC than men but increasing body mass index leads to progressively repressed 4βHC in both sexes.

**What Are the Clinical Implications?**

- PXR‐4βHC‐LXR circuit is a novel blood pressure‐regulating pathway deregulated in overweight and obesity by repressed 4βHC, suggesting a role for plasma 4βHC in overweight‐ and obesity‐induced hypertension.
- As the increasing body mass index is known to be more clearly associated with hypertension in women than in men, the proposed hypotensive effect of 4βHC and the higher plasma 4βHC concentration in lean women than men offer clues to the mechanism of sex‐specific aspects of blood pressure regulation.

Obesity, defined as body mass index (BMI) >30 kg/m^2^, and overweight (BMI, 25–30 kg/m^2^) are worldwide health problems with increasing prevalence. World Health Organization has estimated that >1.9 billion adults globally were overweight and >650 million were obese in 2016.^1^ The age‐adjusted prevalence of obesity increased from 35.4% to 43.4% between 2011 and 2018 in the United States.^2^ Overweight and obesity expose to a wide array of health problems including type 2 diabetes, cardiovascular diseases, cancer, and pulmonary and musculoskeletal diseases. Overweight and obesity also increase blood pressure and are recognized risk factors for hypertension, the number 1 cause for disability and years of life lost globally.^3^ There is a nearly linear relationship between BMI and blood pressure already in normal weight and also in overweight people.^4^ However, the mechanisms mediating the hypertensive effect are partly obscure.^5^

Among the most abundant circulating oxysterols, the oxidation products of cholesterol, is 4β‐hydroxycholesterol (4βHC) formed by cytochrome P450 (CYP) 3A4 and CYP3A5 enzymes in the liver.^6^ CYP3A4 is the major CYP3A enzyme while the polymorphic CYP3A5 is expressed in <10% of White people. A main factor regulating CYP3A expression is pregnane X receptor (PXR, *NR1I2*).^7^ PXR is a xenobiotic‐sensing transcription factor, and its large ligand‐binding pocket accepts a multitude of xenobiotics including environmental toxicants and many therapeutic drugs, with one of the most efficient human agonists being the clinically used antibiotic rifampicin. The activation of PXR increases plasma 4βHC several‐fold through the induction of CYP3A enzymes.^8^ In addition to controlling the detoxification machinery, PXR also regulates glucose metabolism, inflammatory response, and cholesterol synthesis.^9^, ^10^, ^11^

In our prior publication on rifampicin effects on 24‐hour blood pressure (BP)^12^ we demonstrated that rifampicin dosing elevated 24‐hour mean systolic BP (SBP) and DBP (4.7 mm Hg, *P*<0.0001, and 3.0 mm Hg, *P*<0.001, respectively) as well as heart rate (HR). Although rifampicin elevated both 24‐hour BP and 4βHC (>3‐fold as expected), there was an unexpected negative correlation between 24‐hour SBP and plasma 4βHC in both the placebo and rifampicin arms (rifampicin 24‐hour SBP, *r*=−0.69, *P*<0.001; placebo 24‐hour SBP, *r*=−0.70, *P*<0.001).^12^

Thus, PXR and 4βHC axis may constitute a novel mechanism regulating human BP with PXR activation increasing and 4βHC decreasing BP. There is some prior evidence suggesting that PXR activation could have direct vasoactive effects on mice aorta and mesenteric arteries.^13^, ^14^ Regarding 4βHC, we have previously shown that it can signal changes in the hepatic metabolic state to peripheral tissues^15^ by activating liver X receptors (LXR) α an β.^16^ LXR has been implicated in the regulation of BP.^17^ We now provide novel evidence on the BP‐elevating effect of PXR activation and BP‐reducing effect of 4βHC in rats and humans, and demonstrate 4βHC as an independent indicator for BP in a study population consisting of healthy volunteers and obese patients. In the context of prior research on vasoactive functions of LXR, our findings establish PXR‐4βHC‐LXR as a novel pathway of BP regulation.

## Methods

### Study Design of the Rifampicin Clinical Trials and the Cohort Study of Subjects With Obesity

The data that support the findings of this study are available from the corresponding author upon reasonable request. Four previously performed clinical trials exploring metabolic effects of rifampicin on healthy volunteers were used in the current study. Rifa‐1,^18^ Rifa‐BP,^12^ and Rifa‐Stea (unpublished) trials had a randomized placebo‐controlled crossover design with a minimum of 4‐week wash‐out period. The fourth trial (Rifa‐2),^19^ had a 1‐arm design with no control arm. Rifa‐1, Rifa‐2, and Rifa‐Stea had an open design while Rifa‐BP was single‐blind with study personnel blinded. The subjects in all 4 studies were administered 600 mg rifampicin (Rimapen, Orion Inc., Espoo, Finland) once a day for a week. The studies were designed to explore the effects of PXR activation on glucose tolerance (Rifa‐1, n=12), incretin secretion (Rifa‐2, n=12), blood pressure regulation (Rifa‐BP, n=22) and hepatic fat content (Rifa‐Stea, n=16). The inclusion criteria were healthy volunteers with age between 18 and 40 years (upper limit was 45 years in Rifa‐2). The body mass index (BMI) criterion was between 19 and 28 kg/m^2^ in Rifa‐2, 19 to 30 kg/m^2^ in Rifa‐BP, and 18 to 25 kg/m^2^ in Rifa‐Stea. There was no BMI limit in Rifa‐1 study. In Rifa‐BP, inclusion criterion for SBP was 95–140 mm Hg while the other studies did not use BP limits.

The exclusion criteria were any regular medication (hormonal intrauterine device was allowed), any major somatic or psychiatric morbidity (as judged by the study physician on the basis of history, physical examination, and basic laboratory values), insensitivity to rifampicin, continuous use of soft contact lenses (rifampicin may color), pregnancy or breastfeeding, drug or alcohol abuse, history of difficult venipuncture and participation in any other medical study during the study or the past 1 month. Additionally, the DBP >90 mm Hg was an exclusion criterion in Rifa‐BP, and SBP >150 mm Hg in Rifa‐Stea.

The subjects visited the Research Unit of Internal Medicine in the Oulu University Hospital as outpatients. After the first rifampicin tablet was taken under the supervision of a study nurse, the participants were asked to take their daily doses at home between 4 and 8 pm at least 1 hour before and 2 hours after a meal. The details of the experimental protocols in Rifa‐1, Rifa‐2, and Rifa‐BP are described in the previous publications^12^, ^18^, ^19^ The office BP was measured twice with 5‐minute intervals and the average of the 2 measures was calculated in all the trials. For the analyses of this combined study, the values of BP measurements either on the morning of the eighth day (2‐arm Rifa‐1 and Rifa‐Stea studies), on the morning of the first and eighth day (1‐arm Rifa‐2 study), and on the morning of the ninth day (2‐arm Rifa‐BP study) were used as these were the days of blood sampling for the measurements of plasma 4βHC. Also, the fasting weight was measured on these visits without overcoats. The height was measured once during a separate screening visit. The subjects consumed their regular diets during the study arms.

A written, informed consent was obtained from each study subject. The Ethics Committee of the Northern Ostrobothnia Hospital District (Oulu, Finland; decision numbers 78/2009 for Rifa‐1, 73/2010 for Rifa‐2, 6/2012 for Rifa‐BP, and 83/2014 for Rifa‐Stea) and the Finnish Medicines Agency Fimea approved the studies. The study subjects were financially compensated for participation. The trials were registered at https://ClinicalTrials.gov (Rifa‐1 NCT00985270, Rifa‐2 NCT01293422, Rifa‐BP NCT01690104, and Rifa‐Stea NCT02329405).

Also, data from a fifth clinical study was used in the current analysis. The individuals in the gastric bypass surgery study had a medically indicated need for a bariatric surgery.^20^ All participants came for a study visit before the Roux‐en‐Y gastric bypass surgery (RYGB) and gave blood samples. Patients came for a second visit 6 months after the surgery. Inclusion criteria for this study were medical indication for bariatric surgery and age between 18 and 65 years. Exclusion criteria included need for insulin therapy (type 1 or type 2 diabetes) or oral corticosteroids, history of prolonged antibiotic treatments, chronic inflammatory diseases including inflammatory bowel disease and rheumatic diseases, celiac disease, and malignant diseases. Additionally, nondiabetic control individuals with a medical indication for gastroscopy without any suspicion of malignancy were recruited. After the study was stopped because of slow recruitment, there were 34 patients with RYGB and 7 control subjects. There were difficulties in blood draw in 1 individual in the 6‐month visit leading to a lack of 4βHC sample, 3 individuals declined the 6‐month study visit, and 1 individual declined surgery after the first study visit. The results from preoperative (34 individuals) and postoperative periods (29 individuals) as well as 7 control individuals are reported here. The office blood pressure and weight were measured on the visits as described above.

The study protocol was approved by the Ethics Committee of the Northern Ostrobothnia Hospital Districts (decision number 96/2008). All patients provided written informed consent before any study‐related procedure. This study was registered at ClinicalTrials.gov as NCT01330251. The study procedures of all the 5 studies reported here were in accordance with the ethical standards of the Declaration of Helsinki and guidelines on Good Clinical Practice. The demographic parameters of all the study subjects from rifampicin trials and the gastric bypass surgery study are presented in Table S1.

### Effect of PXR Activation on Telemetric Monitoring of Blood Pressure in Rats

Male 2‐month‐old Sprague Dawley rats (240 to 280 g) were obtained from the colony of the Laboratory Animal Centre at the University of Oulu, Oulu, Finland. All rats were kept in plastic cages with free access to tap water and regular rat chow in a room with a controlled 40% humidity and a temperature of 22°C. A controlled 12‐hour environmental light cycle was maintained. The experimental protocols were approved by the Animal Use and Care Committee of the University of Oulu and the Provincial Government of Southern Finland Department of Social Affairs and Health (license number ESAVI‐2010‐07990/Ym‐23). The investigation conforms to the *Guide for the Care and Use of Laboratory Animals* published by the US National Institutes of Health. Animal studies are reported in compliance with the Animal Research: Reporting of In Vivo Experiments guidelines.^21^

The rats were anesthetized with 0.26 mg/kg fentanyl citrate and 8.25 mg/kg fluanisone (Hypnorm; VetaPharma, Leeds, UK), and 4.1 mg/kg midazolam (Dormicum; Roche, Basel, Switzerland) subcutaneously. Antibiotic therapy with 150 mg/kg amoxicillin‐clavulanic acid (Synulox vet, amoxicillin 140 mg/mL clavulanic acid 35 mg/mL subcutaneously; Pfizer Animal Health, Espoo, Finland) was initiated prophylactically 12 hours before the operation against the potential infection and administered twice daily for 3 days after operation. A telemetry device (TA11PA‐C40; Data Sciences International, St. Paul, MN) was implanted in the lower abdominal aorta and secured with surgical glue (3M Vetbond; 3M, St. Paul, MN). Its placement was verified with a radio receiver. Buprenorphine hydrochloride (0.05–0.2 mg/kg subcutaneously; Vetergesic; Orion Pharma, Espoo, Finland,) was administered twice daily for 3 days after operation for postoperative analgesia. BP and HR were continuously measured through the recovery period. Cardiovascular parameters were returning to normal circadian patterns 1 week after implant operation. Following 3 days of baseline recordings, the rats were given single daily intraperitoneal injections of pregnenolone 16α‐carbonitrile (PCN, a rodent PXR agonist, 40 mg/kg; n=8) in corn oil plus 30% dimethyl sulfoxide or vehicle (corn oil plus 30% dimethyl sulfoxide; n=7) for 6 days. In addition to 6‐day dosing period, telemetry recordings were monitored for an additional 8 days. The rats dosed with PCN versus vehicle control are an established model for the study of the effects of PXR activation.^22^ The dosing was randomized; no blinding or sample size calculation was undertaken as those were uncustomary at the time of the study execution. For the same reason, only male rats were used. There were no exclusions of the telemetric data points, and all the animals were followed for the whole timespan planned with no exclusions because of animal welfare or any other reason. The SBP, DBP, and HR were recorded (10‐s telemetered segments were obtained every 3 minutes) and averaged for every hour. Further, all measured variables were again averaged for 12 h and pooled for each day to represent 12‐hour light and dark periods. On completion of the experiment, animals were killed by terminal carbon monoxide anesthesia.

### Laboratory Assays

The clinical laboratory analyses were performed by the Clinical Laboratory of Oulu University Hospital (NordLab, Oulu, Finland) and were validated for clinical use. The photometric enzymatic method was used for plasma creatinine, and the indirect ion selective electrode method for plasma potassium measurements. Photometric method was utilized for the measurement of plasma alanine aminotransferase and alkaline phosphatase. Serum and urine aldosterone were measured with chemiluminescent immunoassay and plasma renin activity was measured with radioimmunoassay. Ultrahigh performance liquid chromatography coupled with high resolution mass spectrometry was utilized as previously described^23^ for the measurement of 4βHC in human plasma at the Admescope Ltd. (Oulu, Finland). The concentration of 4α‐hydroxycholesterol was measured simultaneously to control for the sample storage conditions.^6^ A high‐throughput nuclear magnetic resonance metabolomics platform^24^ was used for the quantification of lipids including the total cholesterol reported here.

Allelic distribution of *CYP3A5* gene SNP 6986A>G (SNP id rs776746) were defined from the rifampicin trial participants (n=62). The SNP was genotyped using Taqman Drug Metabolism Genotyping Assay (Assay ID C_26201809_30) from Thermo Fisher Scientific (Waltham, MA). The assay was performed following manufacturer’s instructions with QuantStudio 5 real‐time PCR system (Thermo Fisher). The assay discriminates alleles 1 (CYP3A5*1) and 3 (CYP3A5*3) using SNP rs776746 (A‐>G change in gene sequence). Nonfunctional/nonexpressor homozygotes (CYP3A5*3/*3) were distinguished from expressor genotypes (homozygotes CYP3A5*1/*1 and heterozygotes CYP3A5*1/*3).^25^ Genotyping was not performed on gastric bypass surgery study as DNA samples were not collected.

### Statistical Analysis

All statistical computations of clinical data were performed using IBM SPSS Statistics for Mac (IBM, Armonk, NY). The study participants and arms were combined for analyses as follows. For the analysis of the effect of rifampicin on the office BP measures, the 4 rifampicin studies on healthy volunteers were combined (n=62) (“PXR activation data set”). As one set of plasma samples for 4βHC analysis was lost, for the correlation of 4βHC with BP and BMI, this data set includes n=61 samples. For the regression analyses of the effect of clinical parameters on the plasma 4βHC, the values before rifampicin dosing (Rifa‐2 study), the values of placebo arm (the other 3 rifampicin studies) and the preoperative and control group values (gastric bypass surgery study) were combined with n=102, referred to as the “non‐intervention data set”. This data set (n=102) was also used to analyze the effect of 4βHC and clinical parameters on the office BP. A flowchart (Figure S1) presents the studies and study arms forming the “PXR activation data set” and the “non‐intervention data set”. As studies designed for other purposes are analyzed here in combined data sets, they represent samples of convenience and, thus, there are no sample size calculations.

Two multiple linear regression analyses were conducted to investigate the effects of various independent variables to our variables of interest; the plasma 4βHC concentration and the SBP. We used backward elimination method for regression to form the model. Exclusion criteria for a variable to be removed from the model were set as *P*>0.7, and for a variable to be considered as a statistically significant, a criterion *P*<0.05 was to be met. Cases were excluded pairwise. During model development, the age was not entered into models for being highly unbalanced between rifampicin trials and RYGB cohort (Table S1) and some parameters such as alanine aminotransferase and BMI correlated too strongly with each other leading to multicollinearity issues.

The effect of rifampicin on BP and other measures were analyzed with paired 2‐tailed Student’s *t*‐test. Pearson correlation coefficients were calculated to analyze the correlation of 4βHC with hemodynamic parameters and BMI. The effect of PCN versus vehicle control on rat hemodynamic parameters was analyzed with 2‐tailed Mann–Whitney *U*‐test. The incremental AUC analyses were performed with GraphPad Prism (GraphPad Software, San Diego, CA). The calculations of incremental AUCs had 3‐day recordings before PCN/vehicle dosing as a baseline.

## Results

### PXR Agonist Rifampicin Elevates Office BP, HR, and Plasma 4βHC in Healthy Volunteers

**Figure 1:**
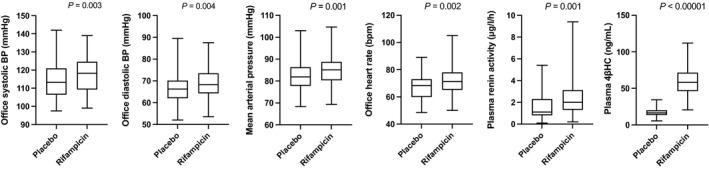
Effect of the treatment with 600 mg rifampicin or placebo once daily for 1 week on blood pressure and blood pressure‐regulating factors in healthy volunteers of the “pregnane X receptor activation data set” (n=62, except for 4β‐hydroxycholesterol analysis n=61). Center line, mean; box limits, upper and lower quartiles; whiskers, minimum and maximum values. 4βHC indicates 4β‐hydroxycholesterol; and BP, blood pressure.

**Figure 2:**
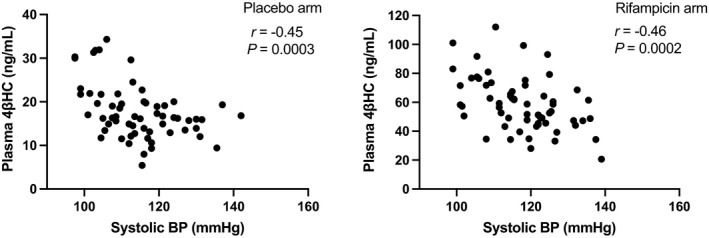
Correlation between plasma 4β‐hydroxycholesterol and systolic blood pressure in the rifampicin and placebo arms in healthy volunteers of the “pregnane X receptor activation data set” (n=61). Pearson correlation coefficients were calculated to analyze the correlation of 4β‐hydroxycholesterol with systolic blood pressure. 4βHC indicates 4β‐hydroxycholesterol; and BP, blood pressure.

Four previously performed clinical trials with rifampicin on healthy volunteers were utilized in the current study. Together, these studies constitute the “PXR activation data set” with n=62 (flowchart as Figure S1). The mean office SBP was elevated by 3.1 mm Hg (*P*=0.003), DBP 1.8 mm Hg (*P*=0.004), mean arterial pressure (MAP) 2.2 mm Hg (*P*=0.001) and HR 4.0 bpm (*P*=0.002) by rifampicin dosing in comparison with placebo (Table S2). Also, plasma renin activity was increased (51%, *P*=0.001). Rifampicin increased plasma 4βHC considerably, as expected (3.3‐fold). Figure 1 visualizes the effects of rifampicin on SBP, DBP, MAP, HR, plasma renin activity and plasma 4βHC levels. There was a negative correlation between the office SBP and the plasma 4βHC in both rifampicin (*r*=−0.46, *P*=0.0002) and placebo arms (*r*=−0.45, *P*=0.0003) (Figure 2). A similar association was detected for MAP and DBP but not for HR (Figure S2). Thus, PXR activation elevates office BP and plasma 4βHC but circulating 4βHC is negatively correlated with office SBP, MAP and DBP in healthy volunteers with normal weight. *CYP3A5* expressors (*1/*3) had higher levels of 4βHC but no genotype effect on BP was detected (Table S3 and Figure S3).

### Plasma 4βHC Is Regulated by Sex, Repressed by BMI, and Is a Predictor for Office BP

**Figure 3:**
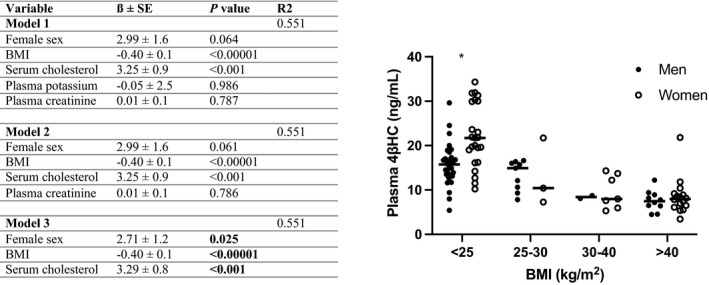
The factors affecting plasma 4β‐hydroxycholesterol concentration. **A**, Predictors for 4β‐hydroxycholesterol in multiple linear regression analysis in the “non‐intervention data set” including healthy volunteers and patients with obesity (n=102). **B**, Individual 4β‐hydroxycholesterol values in 4 body mass index ranges according to sex. Values in the bar graphs are represented as means±SD. *P<0.001, unpaired 2‐tailed Student *t*‐test. 4βHC indicates 4β‐hydroxycholesterol; and BMI, body mass index. Bold indicates statistically significant factors in the final model.

Multiple linear regression analysis of the “non‐intervention data set” including healthy volunteers and patients with obesity (range of BMI, 19.2–55.2 kg/m^2^) demonstrated that serum cholesterol (*P*<0.001) and BMI (*P*<0.00001) are the most significant predictors for plasma 4βHC (Figure 3A). Also, sex affected (*P*=0.025) 4βHC concentration, with women having higher plasma 4βHC than men. However, sex‐difference was only evident in lean subjects as BMI suppressed plasma 4βHC in both sexes at overweight and obese BMI range (Figure 3B). For office SBP, the only statistically significant predictor was plasma 4βHC concentration (*P*=0.020), even when BMI was considered (Tables 1 and 2).

**Table 1:** Predictors for Systolic BP in Multiple Linear Regression Analysis in the “Non‐Intervention Data Set” Including Healthy Volunteers and Patients With Obesity (n=102)

Variable	ß±SE	*P* value	R^2^
Model 1			0.343
Female sex	−5.21±4.2	0.215	
BMI	0.39±0.2	0.059	
4ßHC	−0.61±0.3	0.046	
Plasma creatinine	0.12±0.1	0.387	
Plasma potassium	5.21±6.3	0.408	
Serum cholesterol	−0.79±2.4	0.747	
Model 2			0.342
Female sex	−5.55±4.0	0.177	
BMI	0.38±0.2	0.061	
4ßHC	−0.65±0.3	0.020*	
Plasma creatinine	0.11±0.1	0.408	
Plasma potassium	5.32±6.2	0.396	

4βHC indicates 4β‐hydroxycholesterol; BP, blood pressure; and BMI, body mass index.

*Indicates statistically significant *P* *value*

**Table 2:** Predictors for Systolic BP in Multiple Linear Regression Analysis in the “Non‐Intervention Data Set” Including Healthy Volunteers and Patients With Obesity (n=102) When BMI Is Excluded With the Rationale that BMI Affects the Formation of 4βHC

Variable	ß±SE	*P* value	R^2^
Model 1			0.309
Female sex	−4.03±4.2	0.340	
4ßHC	−0.96±0.3	<0.001	
Plasma creatinine	0.55±0.1	0.68	
Plasma potassium	8.64±6.1	0.163	
Serum cholesterol	−0.15±2.4	0.953	
Model 2			0.309
Female sex	−4.09±4.0	0.315	
4ßHC	−0.96±0.2	<0.0001*	
Plasma creatinine	0.05±0.1	0.683	
Plasma potassium	8.64±6.1	0.160	

4βHC indicates 4β‐hydroxycholesterol; BP, blood pressure; and BMI, body mass index.

* Indicates statistically significant *P* value

**Figure 4:**
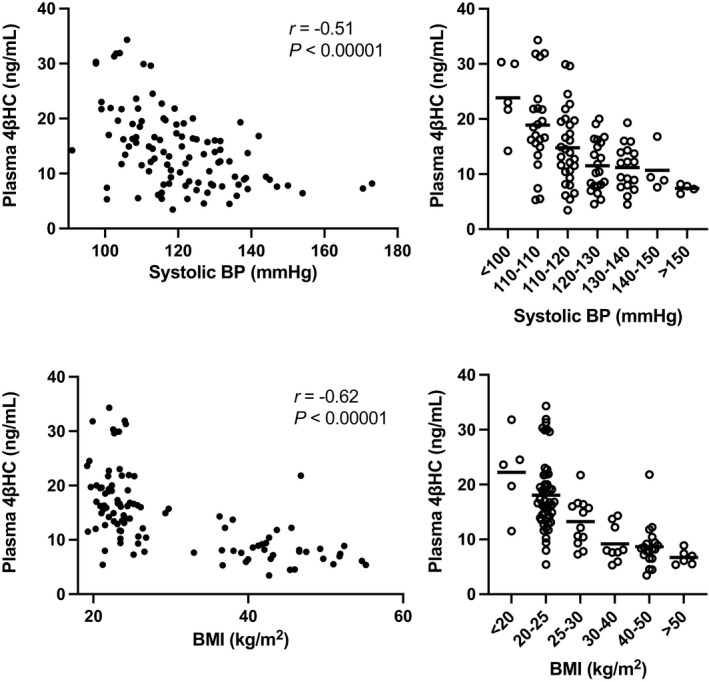
Correlation of plasma 4β‐hydroxycholesterol with (**A**) systolic blood pressure and (**B**) body mass index in the “non‐intervention data set” including healthy volunteers and patients with obesity (n=102). Pearson correlation coefficients were calculated to analyze the correlation of 4βHC with systolic blood pressure and body mass index. Values in the bar graphs are represented as means±SD. 4βHC indicates 4β‐hydroxycholesterol; BP, blood pressure; and BMI, body mass index.

Furthermore, the unadjusted correlation between 4βHC and office SBP in the “non‐intervention data set” consisting of the healthy volunteers and obese patients was negative (*r*=−0.51, *P*<0.00001) (Figure 4A) similar to the “PXR activation data set” with the normal‐weight volunteers. In the “non‐intervention data set”, a similar trend was detected for DBP and MAP (*r*=−0.50 and *r*=−0.54, respectively, *P*<0.00001) and an attenuated trend for HR (*r*=−0.23, *P*=0.02) (Figure S4A through S4D). There was a significant negative correlation between 4βHC and BMI (*r*=−0.62, *P*<0.00001; Figure 4B). It is noteworthy that higher BMI already in the overweight range (BMI, 25–30 kg/m^2^) was associated with reduced mean plasma 4βHC (13.2 versus 18.4 ng/mL, *P*=0.01) when compared with subjects with BMI <25 kg/m^2^ (Figure 4B). The correlations of 4βHC with SBP, MAP, and BMI in all the data sets separately are presented in Table 3. Thus, we demonstrate in healthy volunteers and patients with obesity that plasma 4βHC has significant negative correlation with SBP over a broad range of BMI. Importantly, although overweight and obesity repress plasma 4βHC concentration, 4βHC is a more important predictor of SBP than BMI.

**Table 3:** Correlations of 4βHC With Systolic BP, Mean Arterial Pressure, and BMI

Study arm	No.	Systolic BP		MAP		BMI	
		*r*	*P* value	*r*	*P* value	*r*	*P* value
1) Rifampicin arm	61	−0.46	0.0002	−0.42	0.001	−0.26	0.044
2) Placebo arm	61	−0.45	0.0003	−0.36	0.004	−0.17	0.179
3) Preoperative and controls	41	−0.24	0.138	−0.22	0.161	−0.50	0.001
4) Non‐intervention data set	102	−0.51	<0.00001	−0.54	<0.00001	−0.62	<0.00001

The subsets include rifampicin and placebo arms of the “pregnane X receptor activation data set”, the preoperative period and controls of the gastric bypass surgery study, and the “non‐intervention data set” (healthy volunteers of placebo arms, and the preoperative period and controls of the gastric bypass surgery study; see flowchart in Figure S1). Pearson correlation coefficients were calculated to analyze the correlation of 4β‐hydroxycholesterol with hemodynamic parameters and body mass index. 4βHC indicates 4β‐hydroxycholesterol; BMI, body mass index; BP, blood pressure; and MAP, mean arterial pressure.

### Effect of RYGB Operation on Blood Pressure and Plasma 4βHC Concentration

RYGB operation (n=29) was associated with a reduction in SBP, DBP and HR at 6 months after the operation while plasma 4βHC concentration was not affected (baseline 8.6±3.5 ng/mL; 6 months 9.2±5.1 ng/mL; NS) (Table S4). BMI was reduced by RYGB from the mean of 44.7 to 35.1 kg/m^2^. Even after RYGB, BMI was still in the range of BMI where plasma 4βHC is notably repressed as shown with the preoperative data (Figure 4B). Thus, RYGB data could not be utilized to test the effect of 4βHC modulation on BP regulation.

### PXR Activation Elevates SBP and Elevated 4βHC Decreases SBP and HR in Rats

**Figure 5:**
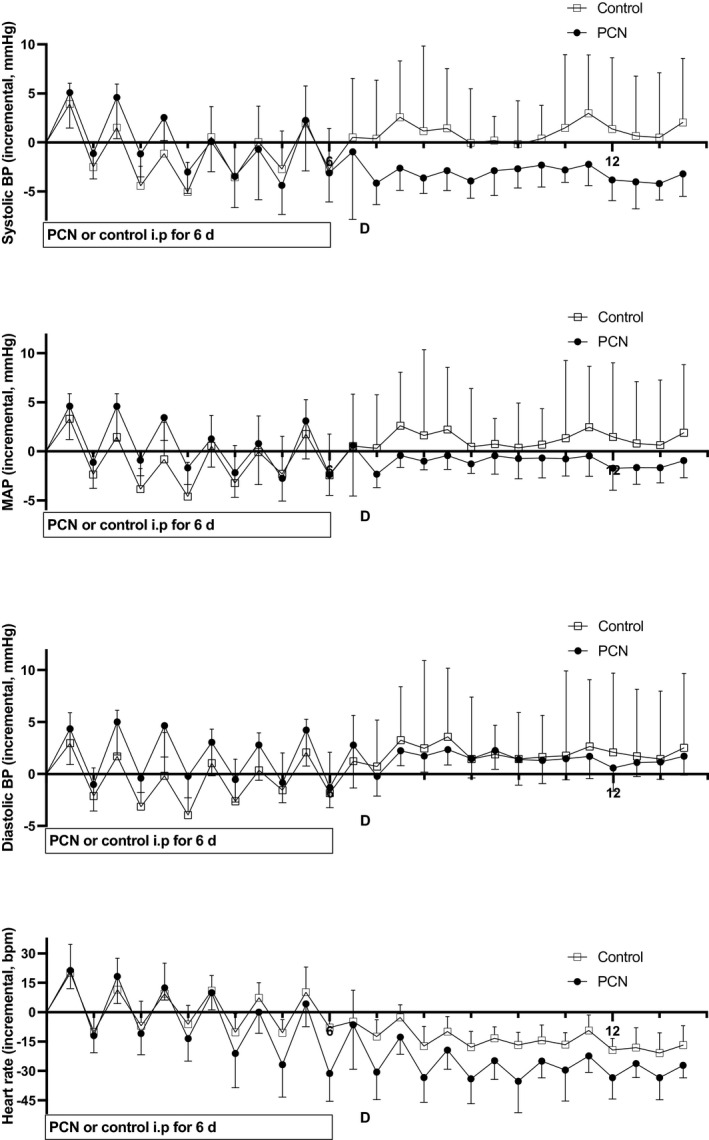
Effect of intraperitoneal pregnenolone 16α‐carbonitrile vs vehicle control for 6 days on incremental systolic blood pressure, mean arterial pressure, diastolic blood pressure, and heart rate in rats followed for total of 14 days. Pregnenolone 16α‐carbonitrile dosing increased systolic blood pressure during the first 3 days (*P*=0.029, see Table 4). After pregnenolone 16α‐carbonitrile was withdrawn and elevated 4β‐hydroxycholesterol was left to act alone (days 7–14), systolic blood pressure and heart rate were lowered (*P*=0.040 and 0.014, respectively, see Table 4). Mann‐Whitney *U* test was the statistical test used. Values are represented as means±SD. 4βHC indicates 4β‐hydroxycholesterol; BP, blood pressure; i.p., intraperitoneal; MAP, mean arterial pressure; and PCN, pregnenolone 16α‐carbonitrile.

To demonstrate that PXR‐4βHC‐LXR circuit is functional in rodents, we performed a proof‐of‐concept experiment where rats were dosed with PCN, a prototypical PXR agonist, versus vehicle control for 6 days. Serum 4βHC is known to be elevated 8.7‐fold in day 6 of PCN 40 mg/kg dosing (Table 4).^15^ The SBP, DBP, and HR were recorded continuously for 14 days and averaged each day to represent 12‐hour light and dark periods (Figure 5). Recorded measures were analyzed with incremental area‐under‐the‐curve (iAUC) analysis after the subtraction of 3‐day baseline values (Table 4). For the first 3 days (0–3 days), PCN dosing elevated SBP iAUC (*P*=0.029), but this effect was attenuated, presumably by increasing 4βHC levels, when SBP iAUC was analyzed for 0 to 6 days. Also, MAP was elevated during the first 3 days with borderline statistical significance (*P*=0.054).

**Table 4:** Effect of Intraperitoneal PCN vs Vehicle Control for 6 Days on the Incremental Area Under the Curves of Hemodynamic Parameters

Variable	D	PCN	Control	*P* value (Mann‐Whitney)	95% CI of difference
Systolic blood pressure iAUC, d*mm Hg	0 to 3	2.94±3.9	−3.55±5.7	0.029*	0.73 to 12.9
	0 to 6	−1.69±11.0	−7.36±14.0	0.397	−11.6 to 20.0
	7 to 14	−22.1±11.0	7.06±38.4	0.040*	−42.7 to −1.28
Diastolic blood pressure iAUC, d*mm Hg	0 to 3	5.14±4.1	−2.13±7.6	0.094	−0.53 to 15.6
	0 to 6	9.11±8.3	−3.94±16.0	0.152	−3.77 to 29.7
	7 to 14	10.4±11.2	14.0±39.6	0.694	−12.8 to 22.8
Mean arterial pressure iAUC, d*mm Hg	0 to 3	3.75±3.4	−3.09±6.6	0.054	−0.40 to 14.1
	0 to 6	2.88±7.8	−6.48±15.0	0.281	−7.81 to 25.3
	7 to 14	−6.82±9.1	8.53±39.9	0.189	−24.2 to 8.70
Heart rate iAUC, d*bpm	0 to 3	5.88±10.6	5.44±28.3	0.867	−25.7 to 24.4
	0 to 6	−22.2±23.7	5.78±44.0	0.189	−64.6 to 8.50
	7 to 14	−188±64.1	−99.8±44.4	0.014*	−151 to −27.0
Serum 4βHC, ng/mL	1	85.1±21.1	66.0±13.9	0.089	−2.80 to 39.4
	6	480±94.6	55.2±5.8	<0.00001*	366 to 511
Serum 4αHC, ng/mL	1	13.9±4.4	12.0±2.3	0.353	−1.87 to 6.10
	6	13.7±2.7	14.0±1.9	0.566	−2.70 to 2.00

The calculations of incremental area under the curve had 3‐day telemetric recordings before pregnenolone 16α‐carbonitrile or vehicle dosing as a baseline. Pregnenolone 16α‐carbonitrile or vehicle was dosed for 6 days and telemetric monitoring of blood pressure was continued for further 8 days. Serum 4β‐hydroxycholesterol and 4α‐hydroxycholesterol concentrations on days 1 and 6 were measured in a separate set of rats as reported earlier.^15^ Values are represented as means±SD and the 95% CI of differences between median of arms. Mann‐Whitney *U* test was the statistical test used. 4αHC indicates 4α‐hydroxycholesterol; 4βHC, 4β‐hydroxycholesterol; iAUC, incremental area under the curve; and PCN, pregnenolone 16α‐carbonitrile.

*P Indicates statistically significant value.

During days 7 to 14, after PCN dosing when serum 4βHC is elevated and PCN is no longer affecting BP, SBP iAUC was significantly lowered in PCN‐dosed rats in comparison with control rats (*P*=0.040). Also, HR was decreased after PCN dosing when serum 4βHC was elevated (days 7–14, *P*=0.014) (Figure 5, Table 4). DBP was not affected significantly at any time window. Thus, PXR activation by PCN elevated SBP in rats, a finding analogous to the hypertensive rifampicin effect in humans. Further, PXR activation‐elevated serum 4βHC lowered SBP in rats, a finding similar to the strong negative association of plasma 4βHC with SBP in humans.

## Discussion

We demonstrate that PXR activation by prototypical PXR agonists, rifampicin in humans and PCN in rats, elevates BP. In healthy volunteers, office SBP and DBP, MAP, and HR as well as plasma renin activity were elevated by rifampicin dosing. In rats, PCN dosing elevated SBP. As the population encounters a multitude of chemicals with PXR‐activating properties including dietary, environmental, and occupational chemicals as well as drugs,^26^, ^27^ this finding may have profound effects on hypertension on global scale.

The expression of PXR has been detected in human and mice aorta and mice mesenteric arteries, and direct vascular PXR‐mediated effects have been described in rodents.^13^, ^14^, ^28^ The dosing of 5β‐dihydroprogesterone, a PXR agonist, for mice enhanced endothelium‐dependent relaxation in mesenteric arteries in wild‐type but not in PXR knockout mice.^13^ This effect was attenuated by CYP epoxygenase inhibitor indicating that PXR‐dependent enhancement of vasorelaxation is mediated in part by epoxygenases able to form epoxyeicosatrienoic acids, known vasodilators.^29^ In PXR wild‐type and knockout mice the administration of another PXR agonist, indole 3‐propionic acid known to be generated by the intestinal microbiota, reduced the endothelium‐dependent vasodilation in isolated and cultured aorta and pulmonary arteries only in wild‐type mice.^14^ In cultured aorta‐derived endothelial cells, PXR agonists PCN and indole 3‐propionic acid lowered endothelial nitric oxide synthase expression, suggesting that the impaired vasodilatation may be mediated by reduced nitric oxide levels. It was speculated that the effects of PXR activation depend on the anatomic site as the endothelium‐produced endothelial nitric oxide synthase is of importance in the aorta and the endothelium‐derived hyperpolarizing factors in mesenteric arteries.^14^ As the blood pressure was elevated in our study, the reduction of the endothelium‐dependent vasodilatation is likely to be a more important PXR‐mediated effect from a systemic point of view than the enhanced relaxation of mesenteric arteries. However, it should be noted that direct vasoactive PXR‐mediated effects have not been studied in humans. It is also of interest that endothelial PXR can be activated by laminar shear stress of blood flow in cell models.^30^

Secondly, we demonstrate that the elevated circulating 4βHC leads to lower SBP and HR in rats when the PXR activator is no longer present and 4βHC is left to act alone. In line with the finding in rats, the higher circulating 4βHC was an independent predictor of lower SBP in humans, over a wide range of BMI from lean to severe obesity. The PXR‐regulated 4βHC is an agonist for LXR α and β.^16^ Incubation of human primary monocyte‐derived macrophages and foam cells with 4βHC was recently shown to induce the efflux of cholesterol and the expression of cholesterol efflux transporters ATP binding cassette transporter A1 and G1, well‐known LXR targets.^15^ It was also recently demonstrated that 4βHC triggers de novo lipogenesis in mouse hepatocytes in vitro and hepatosteatosis in vivo.^31^ The lipogenic effect of 4βHC was mediated by both LXR α and β. Thus, there are prior evidence for the function of PXR‐4βHC‐LXR pathway, but our rat experiment demonstrating the PXR activation‐elevated 4βHC reducing SBP and HR is the first evidence of PXR‐4βHC‐LXR circuit having vasoactive effects. Although acute LXR activation induces renin transcription,^17^ the more chronic activation of LXR by synthetic agonists including T0901317 and GW3965 reduces the experimentally stimulated renin‐angiotensin‐aldosterone system and blood pressure.^32^, ^33^, ^34^, ^35^ Together with our findings, these prior results provide direct evidence for the BP‐lowering effect of LXR activation.

An indirect indication of the significance of circulating 4βHC on blood pressure regulation is the clear and progressive elevation of 4βHC concentration during pregnancy. Plasma 4βHC was 1.4‐, 1.9‐, and 2.3‐fold higher in the first, second, and third trimesters, respectively, compared with non‐pregnant women.^36^ The concentration of 4βHC remains high during immediate post‐partum period (<3 weeks after childbirth)^37^ but returns to a normal level at 4 months after delivery.^38^ Most importantly, serum 4βHC concentration is further elevated by 1.6‐fold in pregnancies complicated with preeclampsia, a condition characterized by high blood pressure and proteinuria precipitated by placental dysfunction, when compared with normal pregnancies.^39^ It is noteworthy that LXR signaling has been implicated in preeclampsia.^40^, ^41^ Thus, elevated 4βHC may help the body to adapt to altered vascular homeostasis of normal pregnancy with further elevation in preeclampsia to counter hypertension. From an evolutionary point of view, if 4βHC is a significant regulator of BP during pregnancy, it would explain why a metabolite of such a long half‐live (about 17 days)^6^ would be involved in BP regulation, usually requiring more rapid response times.

The concentration of 4βHC is higher in women than in men, at least in the premenopausal women studied this far.^6^ In accordance, hypertension prevalence is lower in premenopausal women compared to men of similar age.^42^ However, although women have lower BP in early adulthood, women have steeper BP increases than men leading to similar blood pressure levels in later years, especially after menopause.^43^ In our study, women had higher plasma 4βHC concentration than men but this sex‐difference was only evident in normal‐weight subjects as higher BMI progressively suppressed 4βHC in both sexes. Hypertension is known to be more strongly associated with obesity in women than men, and the increasing BMI is more clearly associated with increased BP in women than in men.^44^ Thus, because of obesity, women lose the presumed protection afforded by female sex hormones. This observation is perhaps explained by obese women losing the advantage of having higher circulating 4βHC than men.

The mechanisms of obesity‐induced hypertension have been suggested to include renin‐angiotensin‐aldosterone system activation mediated by the mechanical compression of kidneys, and sympathetic nervous system activation instigated by hyperleptinemia, hyperinsulinemia and/or obstructive sleep apnea leading to impaired renal natriuresis and vasoconstriction.^5^, ^45^ In our study, RYGB operation of obese patients lowered BP but did not affect 4βHC, impeding our intention of using RYGB cohort to test the effect of 4βHC modulation on BP regulation. Thus, in patients with severe obesity, RYGB operation and the subsequent negative energy balance have 4βHC‐independent effects on BP regulation, via mechanisms mentioned above. Also the non‐alcoholic fatty liver disease is implicated as a risk factor for incident hypertension with increased systemic inflammation, insulin resistance, gut dysbiosis and oxidative stress suggested to play mediating roles in activating sympathetic nervous system and renin‐angiotensin‐aldosterone system.^46^ Non‐alcoholic fatty liver disease and hepatic fibrosis are associated with lower plasma 4βHC.^47^ In our study, the plasma level of 4βHC, produced in liver, was progressively suppressed by increasing BMI already starting in the overweight range. Thus, the repression of circulating 4βHC by obesity provides another plausible explanation on why overweight and obesity are such important risk factors for hypertension. As 4βHC dosing was recently shown to trigger hepatosteatosis in mice,^31^ it is quite logical that hepatic 4βHC formation is downregulated in obesity to prevent further non‐alcoholic fatty liver disease progression.

The polymorphic CYP3A5 enzyme is involved in the synthesis of 4βHC and the concentration of circulating 4βHC increases stepwise with the number of functional CYP3A5*1 alleles.^6^ In a meta‐analysis on the effect of *CYP3A5* genotypes on hypertension and BP, no association was found overall.^48^ As the main factors affecting 4βHC levels are obesity, sex, and exposure to PXR‐activating chemicals, and not the genetic factors, and because BP is affected by salt and alcohol intake, antihypertensives, obesity, and sex among others, it is not surprising that finding consistent associations between *CYP3A* genotypes and BP has proven difficult. However, in White populations, a modest association between CYP3A5*1 expression and lower SBP was detected in a meta‐analysis.^48^ This would be consistent with our finding that higher circulating 4βHC was associated with lower SBP.

Based on our clinical and preclinical experiments, we propose a novel PXR‐4βHC‐LXR pathway where (1) PXR activation elevates BP, and (2) circulating 4βHC lowers blood pressure with LXR mediating the hypotensive effect. In addition to our human (rifampicin) and rat (PCN) findings, the hypertensive PXR effects are supported by previous mouse experiments with PXR wild‐type and knockout mice suggesting reduced nitric oxide as a mediator of impaired vasodilatation.^14^ The importance of 4βHC as a hypotensive factor is supported by strong negative correlations with BP in humans and the lowered blood pressure by elevated 4βHC in rats. Chronic LXR agonism is already known to lead to lower BP in animal experiments.^17^

Rifampicin that we used as a PXR agonist is also an antibiotic. However, antibiotics in general are not considered to affect human BP, although dysbiosis of the gut has been associated with hypertension.^49^, ^50^ Rifampicin is also an inhibitor of organic anion‐transporting polypeptide 1B1.^51^ As the half‐life of rifampicin in induced state is only few hours and BP measurements were performed >12 hours after the last dose, any inhibitory effects of rifampicin are unlikely to affect BP. PCN used in rat experiments is not known to have antibiotic or transporter‐inhibiting effects. Furthermore, although we present evidence in 2 species for vasoactive effects of PXR‐4βHC‐LXR circuit in BP regulation, one of the limitations of our study is that we do not provide direct demonstration on the effect of 4βHC dosing on BP.

In summary, we describe a novel BP‐regulating pathway where PXR activation elevates SBP and the elevated 4βHC on the other hand reduces SBP. Repression of circulating 4βHC in people with overweight and obesity may represent a novel pathophysiological mechanism mediating the overweight‐ and obesity‐induced hypertension, an entity with severe consequences for human health.^5^ The repression of plasma 4βHC by BMI already in the overweight range may explain the known nearly linear relationship between BMI and SBP in normal and overweight people.^4^ In addition, certain sex‐specific aspects of blood pressure regulation such as lower BP levels in younger women may be explained by higher plasma 4βHC in women than men. Circulating 4βHC may also be a part of the circuitry aiding in the adjustment of female body to pregnancy. Our findings open new perspectives for the future hypertension research and a novel way to understand the BP‐regulating pathways in overweight and obesity.

## Sources of Funding

The trials and studies were financially supported by grants from the Diabetes Research Foundation, the Finnish Foundation for Cardiovascular Research, the Northern Finland Health Care Support Foundation, Finnish Government Grants for Health Research, and the Finnish Medical Foundation.

## Disclosures

H.H. was employed by and a shareholder of Admescope at the time of the 4βHC method development and analysis but is currently not employed by or shareholder of Admescope. The other authors declare no competing interests.

## Supporting information

Tables S1–S4
Figures S1–S4
