# Supplementary material for: Pregnane X Receptor‒4β‐Hydroxycholesterol Axis in the Regulation of Overweight‐ and Obesity‐Induced Hypertension
Source: J Am Heart Assoc. 2022 Mar 1;11(6):e023492. doi: 10.1161/JAHA.121.023492 (PMC9075316; doi:10.1161/JAHA.121.023492)
Supplement: Supplementary file 1 — Tables S1–S4 Figures S1–S4 [file JAH3-11-e023492-s001.pdf]

SUPPLEMENTAL MATERIAL

Table S1. Participants of the rifampicin trials and the gastric bypass surgery study. A, Demographic and clinical data of all participants of four separate rifampicin trials Rifa-1, Rifa-2, Rifa-BP, and Rifa-Stea. **B,** Information on the hypertension and diabetes diagnoses as well as smoking status of the study participants

A

**Variable Mean ± SD**	**Overall population**	**Participants of rifampicin trials**		**Participants of gastric bypass surgery study**		
	Men (n=53) Women (n=50) Total (n=103)	Men (n=40)	Women (n=22)	Men (n=11)	Women (n=23)	Controls Men (n=2) Women (n=5)
Age (y)	33.1 ± 13.3	23.4 ± 2.4	24.3 ± 5.3	46.6 ± 9.7	46.7 ± 8.5	49.7 ± 12.7
Weight (kg)	88.7 ± 30.4	75.0 ± 9.0	61.1 ± 5.6	136 ± 14.8	121± 22.5	74.1 ± 22.9
Height (m)	1.72 ± 0.1	1.79 ± 0.1	1.65 ± 0.1	1.77 ± 0.1	1.64 ± 0.1	1.71 ± 0.1
BMI (kg/m^2)	30.2 ± 10.6	23.4 ± 2.2	22.6 ± 2.1	43.6 ± 3.7	44.6 ± 6.1	24.7 ± 4.4
Systolic blood pressure (mmHg)	120.1 ± 15.0	119 ± 9.5	106 ± 5.9	138 ± 14.5	125 ± 12.5	126 ± 27.1
Diastolic blood pressure (mmHg)	72.7 ± 11.1	67.6 ± 8.2	65.1 ± 5.8	84.9 ± 7.1	82.2 ± 8.9	75.4 ± 12.5
Mean arterial pressure (mmHg)	88.5 ± 11.5	84.6 ± 7.6	78.9 ± 5.3	102.7 ± 2.5	96.5 ± 1.9	92.3 ± 16.3
Pulse pressure (mmHg)	47.4 ± 10.6	51.2 ± 8.8	41.3 ± 5.1	53.5 ± 3.8	42.8 ± 1.9	50.7 ± 19.3
Heart rate (bpm)	68.3 ± 10.5	68.7 ± 9.9	65.2 ± 9.8	67.8 ± 10.8	71.5 ± 10.1	66.8 ± 16.1
Serum cholesterol (mmol/l)	3.74 ± 0.7	3.69 ± 0.6	4.18 ± 0.7	3.25 ± 0.6	3.71 ± 0.8	3.76 ± 0.3
Plasma creatinine (µmol/l)	70.9 ± 15.5	81.7 ± 10.7	65.0 ± 9.3	75.1 ± 15.1	55.6 ± 12.5	-
Plasma renin activity (µg/l/h)	-	1.88 ± 1.5	1.22 ± 0.7	-	-	-
Plasma ALT (U/l)	37.8 ± 26.6	21.7 ± 7.8	18.8 ± 6.4	56.8 ± 20.3	34.3 ± 21.4	-
Plasma ALP (U/l)	58.1 ± 25.3	66.5 ± 22.7	59.9 ± 13.8	68.2 ± 23.7	70.8 ± 15.7	-
Plasma potassium (mmol/l)	3.89 ± 0.3	3.85 ± 0.2	3.76 ± 0.2	4.08 ± 0.3	3.96 ± 0.3	-
Plasma 4ßHC (ng/ml)	14.4 ± 7.1	15.4 ± 4.4	22.1 ± 7.1	7.61 ± 2.2	8.84 ± 3.9	14.4 ± 9.0
Plasma 4αHC (ng/ml)	3.92 ± 1.3	3.63 ± 1.4	4.79 ± 1.6	3.83 ± 1.2	3.67 ± 0.7	3.97± 0.8

B

**Variable**	**Overall population (n=103)**			**Participants of rifampicin trials (n=62)**			**Participants of gastric bypass surgery study (n=41)**					
							Participants going to bypass surgery (n=34)			Controls (n=7)		
Hypertension diagnosis	Yes (n=24)	No (n=79)		Yes (n=0)	No (n=62)		Yes (n=23)	No (n=11)		Yes (n=1)	No (n=6)	
DM II diagnosis	Yes (n=19)	No (n=84)		Yes (n=0)	No (n=62)		Yes (n=19)	No (n=15)		Yes (n=0)	No (n=7)	
Smoking	Smokers (n=12)	Non-smokers (n=79)	Ex-smokers (n=11)	Smokers (n=3)	Non-smokers (n=57)	Ex-smokers (n=2)	Smokers (n=9)	Non-smokers (n=16)	Ex-smokers (n=8)	Smokers (n=0)	Non-smokers (n=6)	Ex-smokers (n=1)

Values are represented as means ± SD. BMI indicates body mass index; ALT, alanine aminotransferase; ALP, alkaline phosphatase; 4βHC, 4β-hydroxycholesterol; 4αHC, 4α-hydroxycholesterol.

Table S2. Effect of rifampicin or placebo once daily for 1 week on the hemodynamic and biochemical parameters in the healthy volunteers of the "PXR activation dataset".

Variable	Rifampicin	Placebo	*P* value	Ratio and 95% CI of difference	N
Systolic blood pressure (mmHg)	117 ± 10.1	114 ± 10.3	0.003	1.03 (1.07, 5.10)	62
Diastolic blood pressure (mmHg)	68.5 ± 7.2	66.7 ± 7.4	0.004	1.03 (0.61, 2.98)	62
Mean arterial pressure (mmHg)	84.8 ± 7.1	82.6 ± 7.4	0.001	1.03 (0.99, 3.46)	62
Pulse pressure (mmHg)	49.0 ± 9.3	47.7 ± 9.0	0.17	1.03 (-0.57, 3.14)	62
Heart rate (bpm)	71.5 ± 10.5	67.5 ± 10.0	0.002	1.06 (1.53, 6.51)	62
Plasma renin activity (µg/l/h)	2.46 ± 1.9	1.64 ± 1.3	0.001	1.51 (0.33, 1.31)	46
Plasma creatinine (µmol/l)	73.6 ± 13.3	75.8 ± 13.0	0.010	0.97 (-3.73, -0.53)	62
Serum aldosterone (pmol/l)	418 ± 237	394 ± 278	0.534	1.06 (-50.9, 96.8)	46
Urine aldosterone (nmol/l)	41.6 ± 24.7	33.4 ± 22.2	0.147	1.24 (-3.09, 19.4)	24
Plasma ALT (U/l)	20.4 ± 6.9	20.7 ± 7.4	0.768	0.99 (-2.50, 1.85)	62
Plasma potassium (mmol/l)	3.86 ± 0.2	3.82 ± 0.2	0.143	1.01 (-0.017, 0.11)	46
Plasma 4ßHC (ng/ml)	59.0 ± 18.6	17.7 ± 6.3	<0.00001	3.33 (37.6, 45.0)	61
Plasma 4αHC (ng/ml)	3.80 ± 1.6	4.03 ± 1.6	0.050	0.94 (-0.46, -0.0003)	61

Values are represented as means ± SD, the ratio of arithmetic means, and the 95% confidence interval of differences between mean of arms.

Table S3. Systolic BP, diastolic BP and heart rate tested by *CYP3A5* genotype in the "PXR activation dataset".

Variable	Homozygote *CYP3A5*3/*3* (N=56)			Heterozygote *CYP3A5*1/*3* (N=6)		
	Rifampicin	Placebo	*P* value	Rifampicin	Placebo	*P* value
Systolic blood pressure (mmHg)	118 ± 10.1	115 ± 10.5	0.005	113 ± 10.6	111 ± 8.4	0.428
Diastolic blood pressure (mmHg)	68.8 ± 7.3	67.0 ± 7.5	0.008	66.0 ± 6.5	63.5 ± 6.0	0.254
Heart rate (bpm)	71.7 ± 11.0	67.3 ± 10.1	0.002	69.5 ± 4.1	68.6 ± 8.9	0.669
Plasma 4ßHC (ng/ml)	58.3 ± 2.5	17.1 ± 0.8	<0.00001	65.6 ± 8.4	23.5 ± 2.8	0.001

In both genotypes (*CYP3A5*3/*3*, non-expressors; *CYP3A5*1/*3*, expressors), the systolic BP, diastolic BP and heart rate were affected by rifampicin treatment to a similar degree, but the effect was not statistically significant in heterozygotes due to a small number of subjects. Plasma 4ßHC levels increased during rifampicin arm statistically significantly in both genotypes. There were no statistically significant differences between groups (genotypes) in systolic BP, diastolic BP or heart rate. Plasma 4ßHC levels differed statistically significantly (P = 0.024) between genotypes in placebo arm. No expressor *CYP3A5*1/*1* homozygotes were found. Actual number of participants in plasma 4ßHC analysis is N=55 for homozygotes because of one lacking 4ßHC sample pair. 4βHC indicates 4β-hydroxycholesterol; BP, blood pressure.

Table S4. Clinical parameters of gastric bypass surgery patients before and after the operation (n = 29).

**Variable**	**Preoperative visit 0 mo**	**Postoperative visit 6 mo**	***P* value**
Systolic blood pressure (mmHg)	129.2 ± 14.6	125.3 ± 12.5	0.049
Diastolic blood pressure (mmHg)	82.6 ± 8.1	77.5 ± 9.2	<0.001
Heart rate (bpm)	69.7 ± 9.8	60.4 ± 9.3	<0.0001
BMI (kg/m^2)	44.7 ± 5.6	35.1 ± 5.0	<0.0001
Plasma 4ßHC (ng/ml)	8.63 ± 3.5	9.19 ± 5.1	0.32

4βHC indicates 4β-hydroxycholesterol; BMI, body mass index.

Figure S1. A flowchart on the formation of datasets employed in the analyses. Datasets are based on four separate rifampicin clinical trials (Rifa-1, Rifa-BP, Rifa-Stea, and Rifa-2) on healthy volunteers and one cohort study on patients with obesity and their controls (the characteristics of the trials and cohort are presented below the flowchart). As one set of plasma samples for 4βHC analysis was lost (Rifa-Stea), number of subjects in the "PXR activation dataset" for analyses involving 4βHC is 61 and for other purposes 62. For the same reason, the number of subjects in the "Non-intervention dataset" is 102 (only analyses involving 4βHC were performed in this dataset).

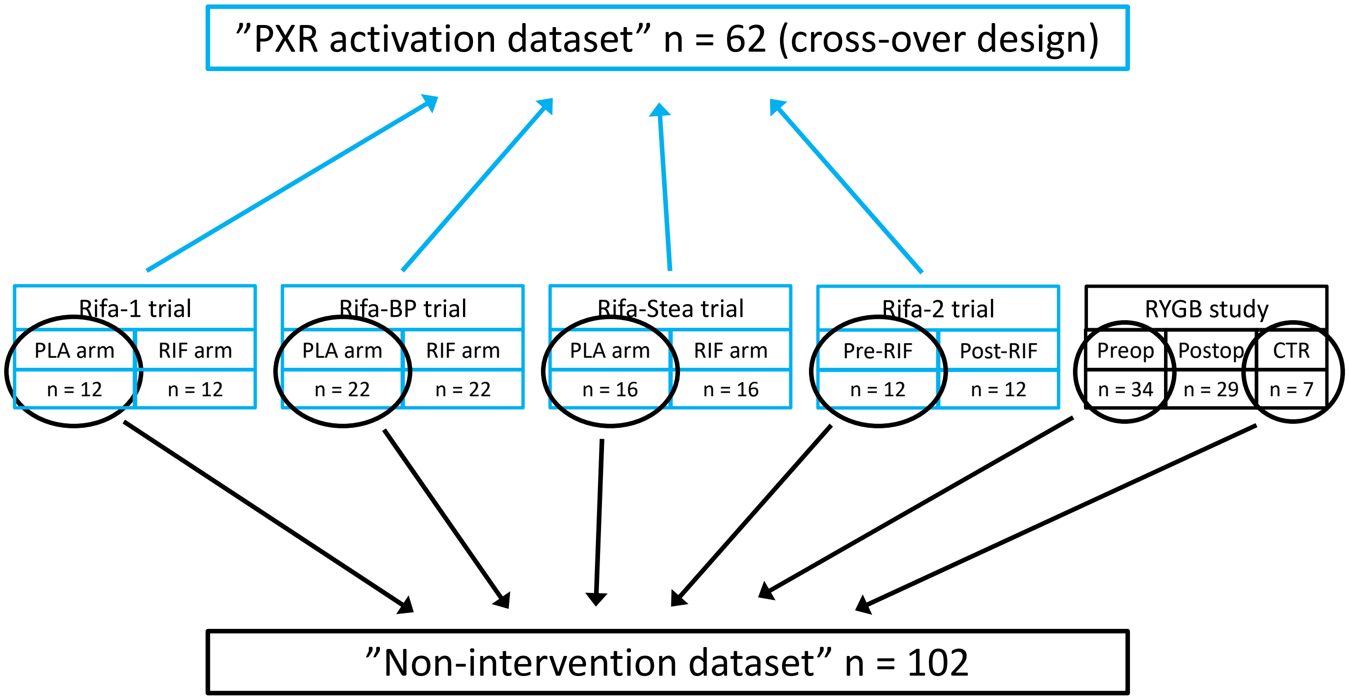

**Variable**	**Rifa-1 Trial**	**Rifa-BP Trial**	**Rifa-Stea Trial**	**Rifa-2 Trial**	**RYGB Study**	
Design	Randomized, crossover, open, placebo-controlled	Randomized, crossover, single-blind (study staff blinded), placebo-controlled	Randomized, crossover, open, placebo-controlled	One-arm study (rifampicin only), open	RYGB surgery	
Study execution	One-week rifampicin arm and one week placebo arm. Visits after each study arm.	One-week rifampicin arm and one week placebo arm. Visits after each study arm.	One-week rifampicin arm and one week placebo arm. Visits after each study arm.	One-week rifampicin arm. Visits before and after study arm.	Preoperative visit before RYGB surgery and postoperative visit after 6 months	
Subjects	Healthy volunteers, all white	Healthy volunteers, all white	Healthy volunteers, all white	Healthy volunteers, all white	Patients with a medically indicated need for bariatric surgery + controls, all white	
					**Patients**	**Controls**
Number of subjects	12	22	16	12	34	7
Mean age ± SD	24.3 ± 5.2 years	24.3 ± 3.5 years	23.2 ± 2.8 years	22.8 ± 3.5 years	46.7 ± 8.8 years	49.7 ± 11.7 years
BMI range	20.4 – 29.3 kg/m^2	20.3 – 29.7 kg/m^2	19.3 – 25.7 kg/m^2	19.5 – 26.1 kg/m^2	36.3 – 55.2 kg/m^2	19.2 – 33.0 kg/m^2
Mean BMI ± SD	23.6 ± 2.7 kg/m^2	23.5 ± 2.1 kg/m^2	22.8 ± 1.9 kg/m^2	22.5 ± 2.0 kg/m^2	44.3 ± 5.4 kg/m^2	24.7 ± 4.0 kg/m^2

BMI indicates body mass index; CTR, controls; PLA, placebo; RIF, rifampicin.

Figure S2. Correlation of plasma 4βHC with (A) mean arterial pressure, (B) diastolic BP and (C) heart rate in the rifampicin and placebo arms in healthy volunteers of the "PXR activation dataset" (n = 61).

A

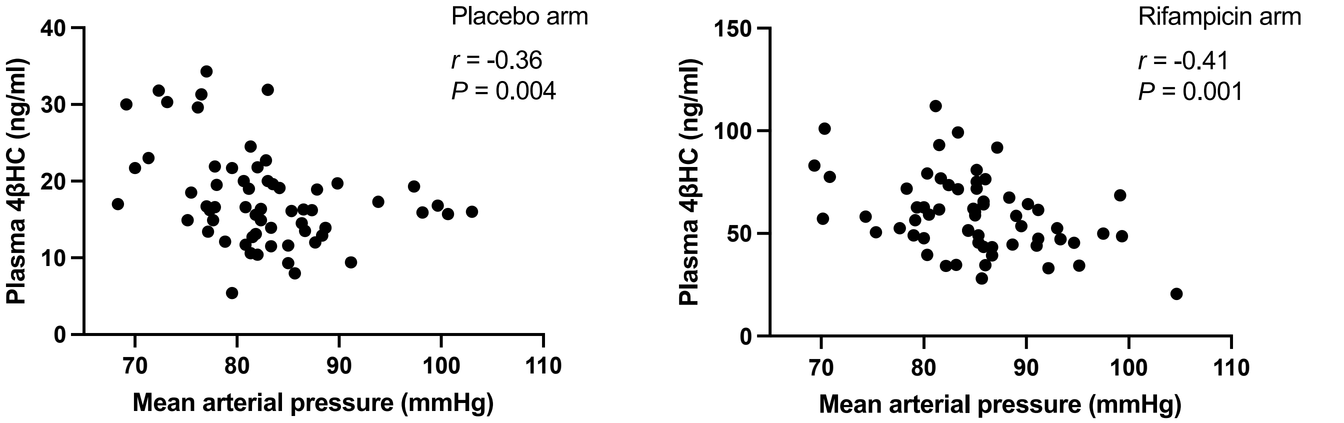

B

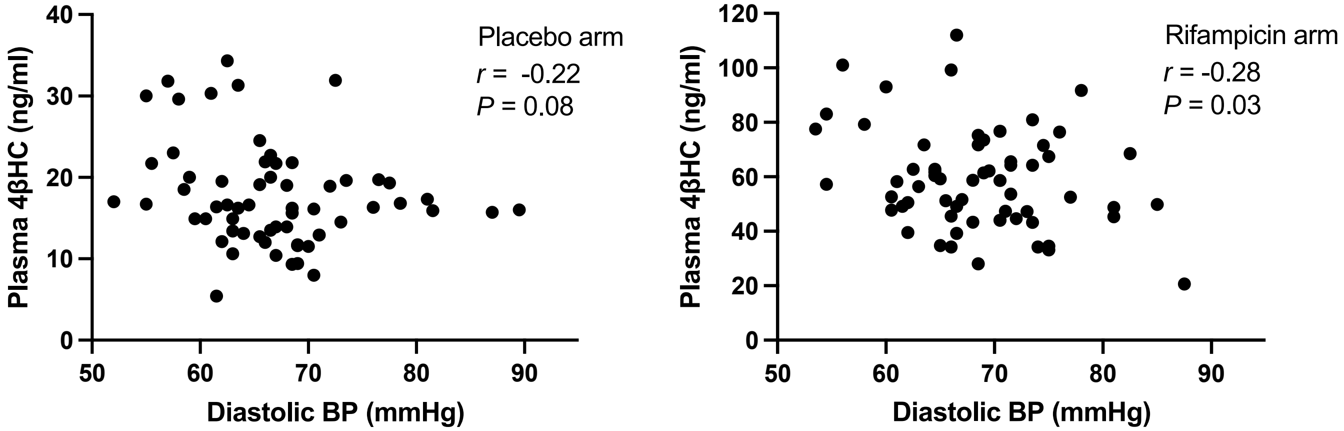

C

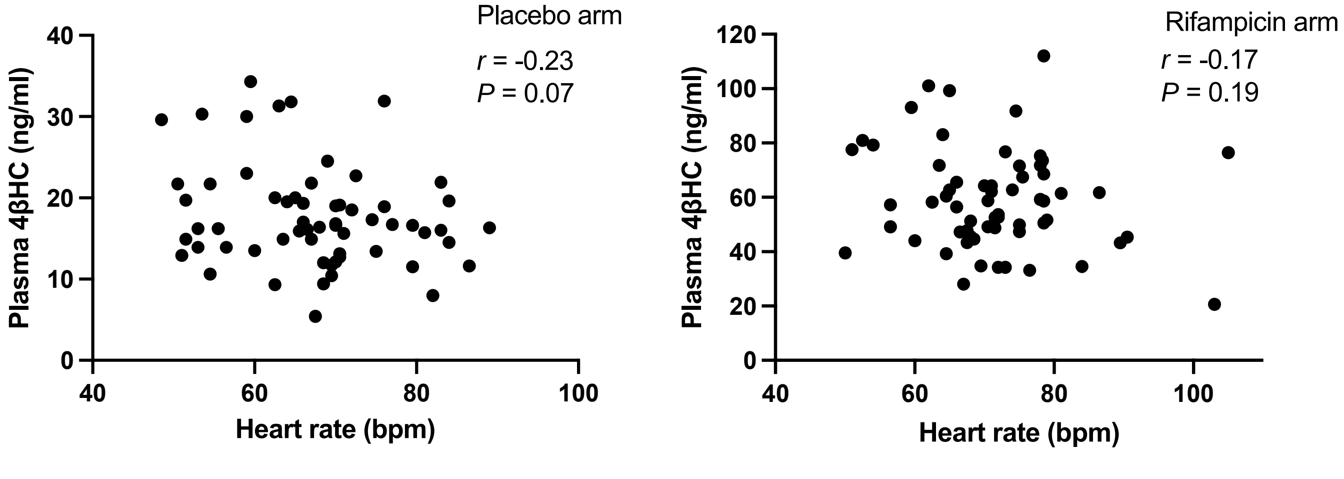

4βHC indicates 4β-hydroxycholesterol; BP, blood pressure

Figure S3. Systolic BP, diastolic BP and HR distribution in rifampicin and placebo study arms according to *CYP3A5* genotype.

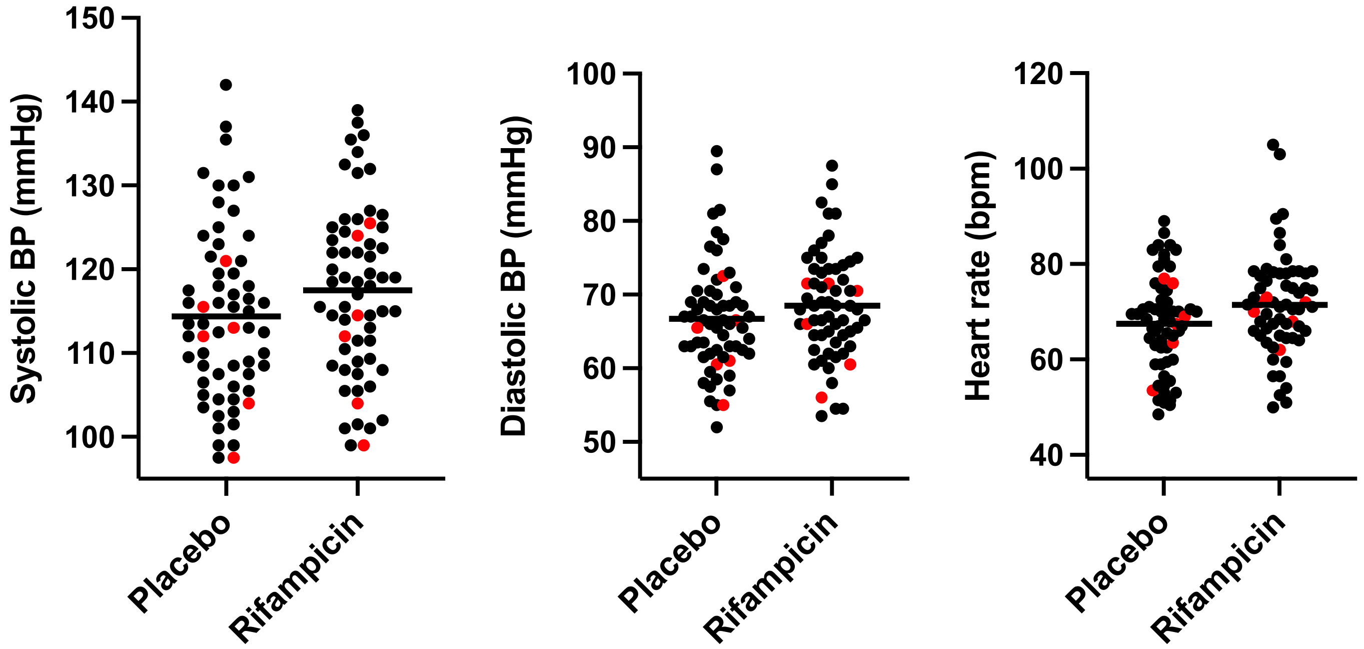

Red spheres represent *CYP3A5*1/*3* heterozygotes (CYP3A5 expressors, n = 6) and black spheres represent *CYP3A5*3/*3* homozygotes (non-expressors, n = 56). BP indicates blood pressure.

Figure S4. Correlation of plasma 4βHC with (A) mean arterial pressure, (B) diastolic BP, (C) heart rate and (D) pulse pressure in the "non-intervention dataset" including healthy volunteers and patients with obesity (n = 102).

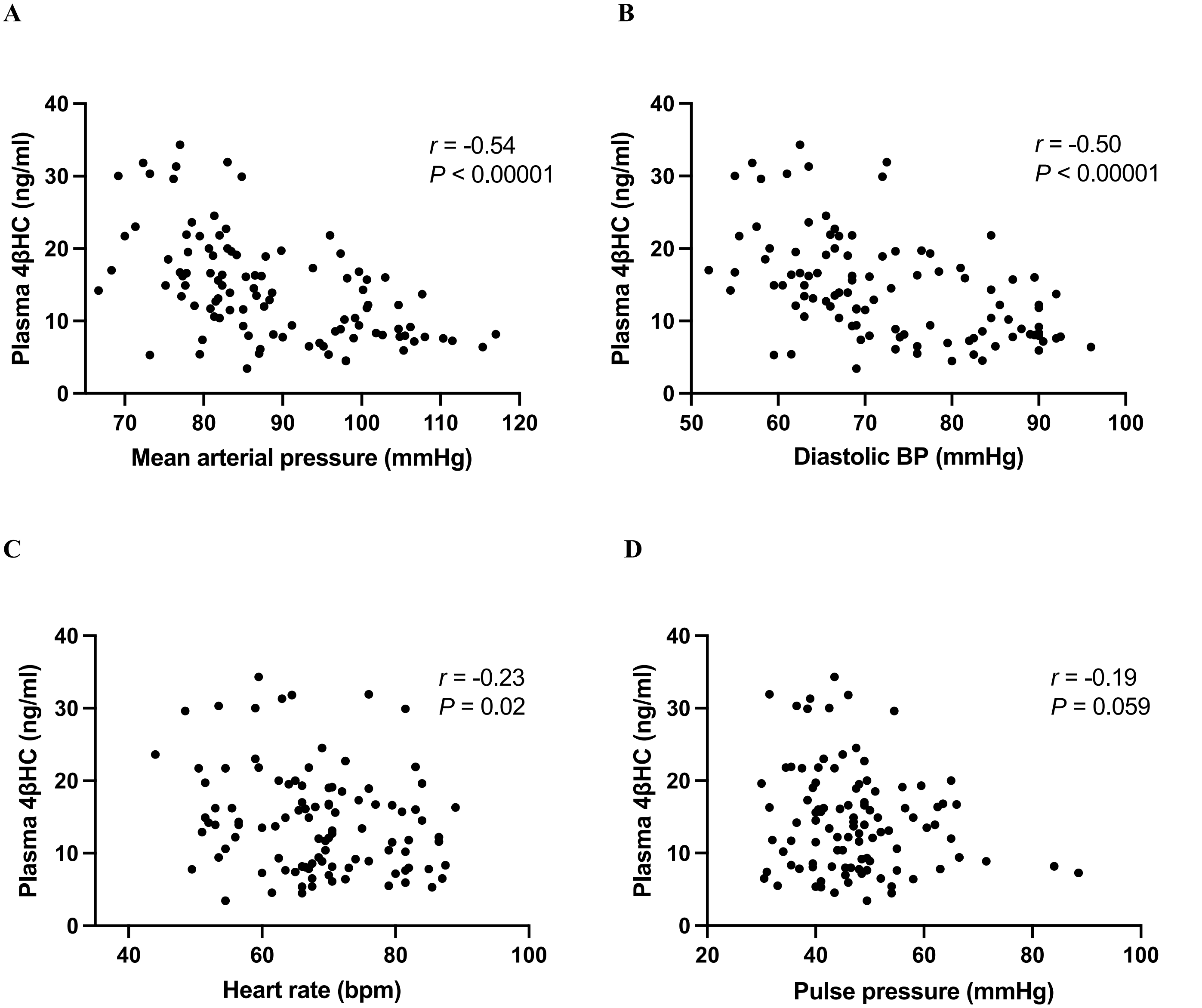

4βHC indicates 4β-hydroxycholesterol; BP, blood pressure.
